# Detection of IgG against *Toxocara* in Sera of Employees of Meat Industry

**Published:** 2015-09

**Authors:** Cosme Alvarado-Esquivel, Jesús Hernández-Tinoco, Luis Francisco Sánchez-Anguiano, Agar Ramos-Nevárez, Sandra Margarita Cerrillo-Soto, Carlos Alberto Guido-Arreola, Leandro Saenz-Soto

**Affiliations:** 1 Biomedical Research Laboratory, Faculty of Medicine and Nutrition, Juárez University of Durango State, Avenida Universidad S/N, 34000 Durango, Mexico;; 2 Institute for Scientific Research “Dr. Roberto Rivera-Damm”, Juárez University of Durango State, Avenida Universidad S/N, 34000 Durango, Mexico;; 3 Clínica de Medicina Familiar, Instituto de Seguridad y Servicios Sociales de los Trabajadores del Estado, Predio Canoas S/N, 34079 Durango, Mexico

**Keywords:** Case-control study, epidemiology, butchers, seroprevalence, toxocariasis

## Abstract

Contact with raw meat could represent a risk for *Toxocara* infection. We assessed the association of *Toxocara* infection with an occupation of meat worker though a case-control seroprevalence study of 124 meat workers and 248 subjects without this occupation. Sera of participants was analyzed for the presence of anti-*Toxocara* IgG antibodies. One (0.8%) of the 124 meat workers, and 5 (2.0%) of the 248 controls were positive for anti-*Toxocara* IgG antibodies (OR=0.39; 95% CI: 0.04-3.41; *P*=0.66). The seropositive meat worker was a male aged 28 years old, without vision impairment. None of the work characteristics i.e. frequency of contact with raw meat, use of safety practices, history of splashes at face with blood or raw meat, and injuries with sharp material at work was associated with *Toxocara* exposure. Seroprevalence of *Toxocara* infection was significantly higher (*P*=0.04) in meat workers with consumption of boar meat (1/6: 16.7%) than in those without this consumption (0/117: 0%). We conclude that meat workers do not have a higher risk for *Toxocara* infection than subjects without this occupation do. The 2% seroprevalence of *Toxocara* infection found in control subjects might suggest a low seroprevalence of this infection among people with other occupations in Durango City. However, additional case-control studies with larger sample sizes to confirm our results are needed.

## INTRODUCTION

*Toxocara* is a nematode parasite that infects cats and dogs throughout the world (1). These animals infected with *Toxocara* shed parasite eggs contaminating the environment (2). Humans become infected with *Toxocara* through ingestion of embryonated eggs or larvae from a range of domestic and wild paratenic animals (3). *Toxocara* disseminates into the bloodstream of infected hosts towards a variety of organs and tissues including eyes, muscles, lungs, liver and central nervous system (2, 4). Most infections with *Toxocara* are asymptomatic (3). However, some infections lead to clinical manifestations, a disease known as toxocariasis. In this zoonotic disease, severe inflammation occurs in major organs or can be limited to eyes and optic nerves (3, 5, 6). Ocular toxocariasis is a cause of blindness (2). Toxocariasis is a neglected zoonosis widely distributed in many countries, the number of cases of toxocariasis is underestimated, and this disease reaches high prevalence independently of socioeconomic conditions (7). Diagnosis of toxocariasis relies upon clinical picture (8) and immunoassays (8, 9). Preventive measures against toxocariasis include deworming pets, sanitary education (9), increasing public awareness and reducing the risk of infection (8). Treatment options for toxocariasis are limited (7). Corticosteroids and diethylcarbamazine (9) or albendazole (8) can be used to treat toxocariasis.

There is increasing evidence that ingestion of raw or undercooked meat, liver or blood from animals infected with *Toxocara* may lead to toxocariasis (10-12). Ocular toxocariasis has been associated with ingestion of raw cow liver or meat in adult patients (13, 14). In addition, consumption of goat meat and raw sea snail was associated with *Toxocara* seropositivity in psychiatric patients in Durango, Mexico (15). Pulmonary toxocariasis has been associated with a history of raw meat intake (16, 17). However, the association of *Toxocara* infection with an occupational exposure to raw meat has been poorly studied. We found only one study about the occupational risk for *Toxocara* infection. In a survey in Austria, the risk for *Toxocara* infection was higher in farmers, veterinarians, slaughterhouse staff and hunters when compared to a control group (18). To the best of our knowledge, there is a lack of information about *Toxocara* infection in meat workers at butcher’s shops. In addition, there is no information about *Toxocara* infection in slaughterhouse workers in Mexico. Therefore, we performed a case-control seroprevalence study to determine whether *Toxocara* infection is associated with an occupation of meat worker in the northern Mexican city of Durango. We also attempted to determine the association of *Toxocara* exposure with the characteristics of meat workers.

## MATERIALS AND METHODS

### Selection and description of participants

Through a case-control study using serum samples from recent *Toxoplasma gondii* serosurveys (19, 20) we studied 124 meat workers and 248 control subjects. Cases and controls were compared for the presence of anti-*Toxocara* IgG antibodies. Inclusion criteria for the meat workers were: 1) currently working as butchers in abattoirs or butcher’s shops for at least 6 months; 2) aged 16 years and older; and 3) who accepted to participate in the study. Gender, socio-economic status, and educational level were not restrictive criteria for enrollment. Meat workers were enrolled in abattoirs, and private butcher’s shops in Durango City, Mexico. Workers included 21 females and 103 males aged 16-71 years old (mean 38.5 ± 13.2 years). Of the 124 meat workers studied, 59 were enrolled in 35 private butcher’s shops, 35 in a federal abattoir and 30 in a municipal abattoir. Controls were selected randomly. Inclusion criteria for controls were: 1) people of the general population of Durango City with occupations other than meat worker; 2) aged 16 years and older; and 3) who accepted to participate in the study. Controls were matched with meat workers by age and gender. We included two controls for each case. The control group included 248 subjects (42 females and 206 males) aged 39.18 ± 13.67 years (range: 16-71 years). The mean age in controls was similar to that in meat workers (*P*=0.50).

### Socio-demographic, clinical, work, and behavioral characteristics of meat workers

A standardized questionnaire was used to obtain the characteristics of the meat workers. Socio-demographic items included gender, age, educational level, and residence, and type of flooring at home. Work items included seniority in the activity, frequency of contact with raw meat, habitual use of safety practices (use of hand gloves and face masks), history of splashes at face with blood or raw meat, injuries with sharp material at work, eating when working, contact with cats or dogs, and cleaning feces. Clinical items were current suffering from any disease, and presence of visual impairment. Behavioral items included contact with animals, foreign traveling, consumption of meat (pork, beef, goat, lamb, boar, chicken, turkey, pigeon, duck, rabbit, venison, squirrel, horse, opossum, or other), and degree of meat cooking.

### Technical information

Sera from cases and controls were kept frozen at –20ºC until analyzed. A commercially available enzyme immunoassay “*Toxocara”* kit (Diagnostic Automation, Inc. Calabasas, CA, U.S.A.) was used to analyze serum samples for anti-*Toxocara* IgG antibodies. We performed all tests following the manufacturers’ instructions. The cut off for seropositivity was an absorbance reading ≥ 0.3 optical density units. Negative and positive controls were included in each run.

### Statistics

Data was analyzed with the aid of the software Epi Info version 7, and SPSS version 15.0 (SPSS Inc. Chicago, Illinois). For calculation of the sample size, we used a 95% confidence level, a power of 80%, a 1:2 proportion of cases and controls, a reference seroprevalence of 13% (21) as the expected frequency of exposure in controls, and an odds ratio of 2.3. The result of the sample size calculation was 111 cases and 222 controls. The paired student’s *t* test was used to compare age values among cases and controls. We assessed the association of *Toxocara* seropositivity with the occupation of meat worker and the socio-demographic, work and clinical characteristics of workers with the two-tailed Fisher’s exact test. The association of *Toxocara* seropositivity with the characteristics of the meat workers was further analyzed by multivariate analysis. Only variables with a *P* value ≤0.15 obtained in the bivariate analysis were analyzed by multivariate analysis. Odds ratio (OR) and 95% confidence interval (CI) were calculated, and statistical significance was set at a *P* value < 0.05.

### Ethical aspects

We studied only archival serum samples and questionnaires from previous surveys in meat workers (19) and general population (20). In these studies, the purpose and procedures of the investigations were explained to all participants and a written informed consent was obtained from all of them and from the next of kin of minor participants. Institutional Ethical Committees approved both previous studies. Furthermore, the ethics committee of the Instituto de Seguridad y Servicios Sociales de los Trabajadores del Estado in Durango City approved this project.

## RESULTS

One (0.8%) of the 124 meat workers, and 5 (2.0%) of the 248 controls were positive for anti-*Toxocara* IgG antibodies (OR=0.39; 95% CI: 0.04-3.41; *P*=0.66). The meat worker seropositive for *Toxocara* had a high anti-*Toxocara* IgG antibody level (optical density units = 2.269). Whereas none of the controls had high (optical density units >2.00) anti-*Toxocara* IgG antibody levels (optical density units in their tests ranged from 0.355 to 1.137). Frequency of high antibody levels in cases was similar (0.8%) to that (0.0%) in controls (*P*=0.33). Of the sociodemographic characteristics, the seropositive meat worker was a male aged 28 years old, born in Durango and had a low socioeconomic status. As to clinical characteristics, this worker was apparently healthy and had no vision impairment. Of the work characteristics, the seropositive meat worker did not use safety practices (gloves or facemask), and did not eat raw meat while working. None of the work characteristics including seniority in the activity, frequency of contact with raw meat, habitual use of safety practices, history of splashes at face with blood or raw meat, injuries with sharp material at work, eating when working, contact with cats or dogs, and cleaning feces was associated with *Toxocara* exposure. With respect to behavioral characteristics, the seropositive meat worker had raised farm animals and had not traveled. He habitually ate well-done meat. Seroprevalence of *Toxocara* infection was higher (*P*=0.04) in meat workers with consumption of boar meat (1/6: 16.7%) than in those without this consumption (0/117: 0%). Consumption of meat from pigeon and squirrel showed *P* values ≤0.15 in the bivariate analysis. Consumption of meats other than those from boar, pigeon, or squirrel showed *P* values higher than 0.15 by bivariate analysis. Multivariate analysis of behavioral characteristics with *P* values ≤0.15 obtained by bivariate analysis showed that none of the three variables (consumption of meat from boar, pigeon and squirrel) assessed was associated with *Toxocara* exposure.

## DISCUSSION

A growing body of evidence indicates that consumption of raw meat is a cause of toxocariasis. *Toxocara* has been found in several animal species slaughtered for human consumption. *Toxocara* larvae have been detected in liver (22) and brain of chickens (22, 23). In addition, *Toxocara* larvae remained infective after refrigeration at 4°C for 28 days in muscles tissues of chickens (24), and were found in liver, muscles and lungs of chickens for half a year (25). In a study in Norway, *Toxocara* was detected in slaughterhouse pigs (26). Furthermore, a number of case reports has described the link between toxocariasis and consumption of raw meat. Consumption of raw chicken meat resulted in visceral toxocariasis (23), and toxocariasis occurred in some family members who ate raw chicken livers (27). Consumption of raw duck liver was linked to cerebral toxocariasis in a 55-year-old woman (28), and a case of neurotoxocariasis was reported in a 51-year-old male who habitually ate raw goat meat (29). Similarly, myocarditis associated with toxocariasis was reported in a 19-year-old man who ate raw deer meat (30), and a 26-year-old man who ate raw meat several times before his hospital admission (31). An increased risk of toxocariasis was related to a recent history of eating raw cow liver (32). Pulmonary toxocariasis was found in a 30-year-old man who often ate raw beef liver (33), and in a 21-year-old woman who ate raw meat and cattle liver (34). *Toxocara* infection in a patient with urticaria and a history of eating raw cattle meat was reported (35). In spite of the reportedly association between toxocariasis and consumption of raw meat, the epidemiology of *Toxocara* infection in workers occupationally exposed to raw meat is largely unknown. The present study aimed to determine the association of *Toxocara* infection with an occupational exposure to raw meat. We found that meat workers had a similar seroprevalence of *Toxocara* infection to people without this occupation. The very low (0.8%) seroprevalence of *Toxocara* infection in meat workers suggests that these workers do not have an important risk for *Toxocara* infection. The lack of association between *Toxocara* seropositivity and the occupational exposure to raw meat was unexpected. Many of the meat workers studied did not use any safety practice to avoid *Toxocara* infections. Some meat workers had had injuries with sharp material and/or splashes at face with blood or raw meat. Furthermore, many butchers had handled meat from several animal species. It is likely that the raw meat handled by the meat workers was from animals without *Toxocara* infection. Consumption of meat does not always represent a risk for *Toxocara* infection. In a study of children in the Republic of the Marshal Islands, consumption of raw meat was not associated with *Toxocara* infection (36). In a study of waste pickers in Durango City, Mexico, consumption of chicken meat was negatively associated with *Toxocara* infection (37). Freezing inactivates *Toxocara* (38). Therefore, it is also likely that handling of frozen meat by butchers might have reduced the risk for *Toxocara* infection. The seroprevalence found in meat workers is lower than seroprevalences of *Toxocara* infection reported in several population groups in the region including 13% in waste pickers (37), 4.7% in psychiatric patients (39), and 26.2% in rural Tepehuanos (40). In addition, the seroprevalence found in meat workers is comparable to those (1.8%-2.5%) found in patients with vision impairment (41), schizophrenic patients (11), and gardeners in Durango City (42). Altogether, results indicate that meat workers do not represent a risk group for *Toxocara* infection.

We searched for contributing factors for *Toxocara* infection in meat workers. However, the low frequency of *Toxocara* exposure found did not allow us to reach statistically significant associations between *Toxocara* exposure and sociodemographic, clinical, work and behavioral characteristics of meat workers.

## CONCLUSIONS

Results indicate that meat workers do not have a higher risk for *Toxocara* infection than subjects without this occupation do. However, additional case control studies with larger sample sizes are needed to confirm or challenge the lack of association of toxocariasis with a meat worker occupation.
